# Comparative evaluation of early diabetic outcomes in southeast asian patients with type 2 diabetes mellitus undergoing Roux-en-Y gastric bypass (RYGB) versus sleeve gastrectomy (LSG)

**DOI:** 10.1038/s41598-024-51384-1

**Published:** 2024-01-05

**Authors:** Wei Soon Pang, Guo Hou Loo, Guo Jeng Tan, Mardiana Mardan, Reynu Rajan, Nik Ritza Kosai

**Affiliations:** 1 Department of Surgery, Pusat Perubatan Universiti Kebangsaan Malaysia, 56000 Kuala Lumpur, Malaysia; 2 Department of Internal Medicine, Pusat Perubatan Universiti Kebangsaan Malaya, 59100 Kuala Lumpur, Malaysia

**Keywords:** Endocrine system and metabolic diseases, Medical research, Obesity

## Abstract

Obesity and type 2 diabetes mellitus (T2DM) is an alarming problem globally and a growing epidemic. Metabolic surgery has been shown to be successful in treating both obesity and T2DM, usually after other treatments have failed. This study aims to compare Roux-Y gastric bypass and sleeve gastrectomy in determining early diabetic outcomes in obese Malaysian patients with T2DM following surgery. A total of 172 obese patients with T2DM who were assigned to either laparoscopic Roux-en-Y gastric bypass (LRYGB) or laparoscopic sleeve gastrectomy (LSG) were analysed up to a year post-procedure. The patients’ T2DM severity were stratified using the Individualized Metabolic Surgery (IMS) score into mild, moderate and severe. Remission rates of diabetes were compared between surgical techniques and within diabetic severity categories. T2DM remission for patients who underwent either surgical technique for mild, moderate or severe disease was 92.9%, 56.2% and 14.7% respectively. Both surgical techniques improved T2DM control for patients in the study. Comparing baseline with results 1 year postoperatively, median HbA1c reduced from 7.40% (IQR 2.60) to 5.80% (IQR 0.80) (*p* < 0.001), mean total antidiabetic medications use reduced from 1.48 (SD 0.99) to 0.60 (SD 0.86) [*p* < 0.001], insulin usage reduced from 27.9 to 10.5% (*p* < 0.001), and T2DM control improved from 27.9 to 82% (*p* < 0.001). The patients had a median excess BMI loss of 69.4% (IQR 34%) and 53.2% (IQR 36.0%) for RYGB and SG respectively (*p* = 0.016). At one year following surgery, there is no difference between LRYGB and LSG in terms of diabetic remission. LSG is not inferior to LRYGB in terms of early diabetic outcomes. Milder T2DM shows a better response. LSG is a simpler procedure with a lower risk profile and should be considered as an early treatment option for obese patients with T2DM.

## Introduction

Diabesity is a term that describes the combination of obesity and Type 2 diabetes mellitus^1^. Nearly a fifth of the Malaysian population today is either diabetic, obese, or both. About 30% of Malaysians are overweight, and a further 20% are obese^2^. The prevalence of T2DM amongst the Malaysian population has almost tripled since year 1980^3^. In spite of this, Malaysians are neither aware nor concerned about the consequences.

Currently, bariatric-metabolic surgery (BMS) is a recommended treatment option for Asians with T2DM with BMI ≥ 32.5 kg/m^2^ who do not achieve durable weight loss and improvement of co-morbidities via non-surgical methods. It is usually considered only after earlier lifestyle and pharmacological interventions have failed^4^. This inadvertently risks the procedure being done in patients with more advanced diabetes for which the benefit of BMS would be reduced.

The two most common BMS procedures today in Malaysia are the combined restrictive-malabsorptive laparoscopic Roux-en Y gastric bypass (LRYGB), and the purely restrictive laparoscopic sleeve gastrectomy (LSG). The choice of procedure is largely up to the surgeon and the patient. LSG is a more suitable option in patients with worse co-morbidities, poorer functional status, difficulty to attend follow up and inadequate operation theater time, but is contraindicated in those with severe gastro-oesophageal reflux disease. Although both procedures lead to weight loss and favourable glycemic outcomes, LRYGB was found to be more superior in terms of weight loss^5^.

There are no objective methods to help determine the preferred BMS procedure for obese diabetic Malaysians. We aim to investigate the importance of procedural selection by reviewing differences in diabetic outcomes of LRYGB and LSG in obese Malaysians with T2DM at 1-year following surgery.

## Results

### Study population profile

The baseline characteristics of all the patients are summarised in Table 1. The study cohort had a mean age of 43.95 (SD 9.52) years and 64.5% were female. Patients had a median diabetic duration of 3 (IQR 7) years, with 27.9% using insulin. Median HbA1c was 7.40% (IQR 2.6), and 27.9% had adequate diabetic control (HbA1c ≤ 6.5) and median BMI was 40.7 kg/m^2^ (IQR 12.4). Of the 172 patients, 72 (41.9%) underwent LRYGB and 100 (58.1%) underwent LSG.

**Table 1:** Pre and postoperative characteristics of obese Malaysians with T2DM undergoing BMS n = 172.

Diabetic severity category, n	Pre op	1 year Post op	*P*^a^
Mild	44 (24.4%)		
Moderate	96 (55.8%)		
Severe	34 (19.8%)		
Race, n			
Malay	137 (79.7%)		
Chinese	10 (5.8%)		
Indian	21 (12.2%)		
Other ethnicity	4 (2.3%)		
Age, years	43.95 (SD 9.52)		
Female	111 (64.5%)		
Duration of diabetes, years	3 (IQR 7)		
BMI, kg/m^2^	43.3 (IQR 12.4)	31.7 (IQR 9.1)	< 0.001
Number of medications, mean	1.48 (SD 0.99)	0.60 (SD 0.86)	< 0.001
HbA1c, %	7.40 (IQR 2.60)	5.80 (IQR 0.80)	< 0.001
Insulin use, n	48 (27.9%)	18 (10.5%)	< 0.001
Glycemic control (HbA1c ≤ 6.5), n	48 (27.9%)	141 (82.0%)	< 0.001

BMI: Body mass index, ^a^Wilcoxon signed rank test, McNemar test.

The baseline characteristics of these patients within their respective surgical techniques are described in Table 2. When broken into their respective T2DM severity category using the IMS score, both groups had an almost equal number of mild T2DM patients. There were more moderate T2DM patients in the LSG group while the LRYGB group had more severe T2DM patients. Median HbA1c was higher in the LRYGB group at 8.05% (IQR 3.5) when compared to 7.20% (IQR 2.3) in the LSG group, while patients with controlled T2DM (HbA1c ≤ 6.5%) constituted 26% and 29% of each surgical technique groups respectively.

**Table 2:** Preoperative characteristics of patients according to surgical technique.

	**RYGB**	**SG**	**P**^**a**^
Count, n	72 (41.9%)	100 (58.1%)	
T2DM severity, n			
Mild	20 (27.8%)	22 (22%)	0.002
Mod	30 (41.7%)	66 (66%)	
Severe	22 (30.6%)	12 (12%)	
Race, n			
Malay	56 (77.8%)	81 (81%)	0.189
Chinese	2 (2.8%)	8 (8%)	
Indian	11 (15.3%)	10 (10%)	
Other ethnicities	3 (4.2%)	1 (1%)	
Age, years	45.11 (SD 8.44)	43.08 (SD 10.21)	0.175
Female, n	60% (43)	68% (68)	0.332
Duration of diabetes, years	4.5 (IQR 9.0)	2.0 (IQR 5.0)	0.011
BMI, kg/m^2^	40.7 (IQR 10.6)	45.2 (IQR 12.7)	0.006
Number of medications, n	1.69 (SD 1.16)	1.0 (SD 0.83)	0.031
Insulin use, n	28 (39%)	20 (20%)	0.009
HbA1c, %	8.05 (IQR 3.50)	7.20 (IQR 2.30)	0.194
Glycemic control (HbA1c ≤ 6.5)	19 (26%)	29 (29%)	0.734

RYGB: Roux-Y gastric bypass, SG: sleeve gastrectomy, BMI: Body mass index, ^a^independent t-test, Chi^2^, Mann–Whitney U test.

The patients in the LRYGB group had more severe T2DM, with a median T2DM duration of 4.5 years (IQR 9.0), and a mean number of anti-diabetic medications of 1.69 (SD 1.16), with 39% of them requiring insulin. Patients in the LSG group were heavier with a median BMI of 45.2 kg/m^2^ (IQR 12.7) versus 40.7 kg/m^2^ (IQR 10.6) in the LRYGB group.

### Results at 1-year post-surgery

T2DM improved regardless of surgical technique, as seen in the changes of HbA1c level, number of medications required, insulin use and disease control (Table 1). 1 year after surgery, disease control increased from 27.9 to 82% (*p* < 0.001), insulin use decreased from 27.9 to 10.5% (*p* < 0.001), and the mean number of anti-diabetic medication reduced from 1.48 (SD 0.99) to 0.60 (SD 0.86) (*p* < 0.001).

There was no difference when comparing diabetic outcomes between LRYGB and LSG. Diabetic remission was 54.2% for LRYGB and 59% for LSG. Both procedures also had almost equal values for improvement and unchanged outcome measures (*p* = 0.804). There was no statistical difference between LRYGB and LSG for reducing HbA1c by 1.80% (IQR 3.30) and 1.50% (IQR 1.80) respectively, *p* = 0.324. Both procedures also led to a reduction in the mean number of anti-diabetic medications by 1.07 (SD 1.17) and 0.73 (SD 0.81) for RYGB and SG respectively, *p* = 0.095. Glycemic control was increased in both procedures to 79.2% and 84% respectively, *p* = 0.429. Although postoperative insulin use showed a statistically significant difference between the two procedures, this cannot be concluded as a true difference as LRYGB had significantly more insulin users than the LSG group (Table 3). With regards to weight loss, LRYGB induced a greater excess BMI loss (EBMIL) of 69.4% [IQR 34.0] when compared to LSG at 53.2% [IQR 36.0], *p* = 0.016 (Table 4).

**Table 3:** Diabetic performance of patients according to surgical technique.

	RYGB	SG	P^a^
Diabetic outcome*, n			
Remission	39 (54.2%)	59 (59%)	0.804
Improvement	24 (33.3%)	29 (29%)	
Unchanged	9 (12.5%)	12 (12%)	
HbA1c, %			
Baseline	8.05 (IQR 3.50)	7.20 (IQR 2.30)	0.194
Postoperative	5.80 (IQR 1.0)	5.80 (IQR 0.8)	0.727
Change	1.80 (IQR 3.30)	1.50 (IQR 1.80)	0.324
P^b,#^	< 0.001	< 0.001	
Number of medications, n			
Baseline	1.69 (SD 1.16)	1.0 (SD 0.83)	0.031
Postoperative	0.62 (SD 0.81)	0.59 (SD 0.89)	0.545
Change	1.07 (SD 1.17)	0.73 (SD 0.81)	0.095
P^b,#^	< 0.001	< 0.001	
Insulin use, n			
Baseline	28 (39%)	20 (20%)	0.009
Postoperative	12 (16.7%)	6 (6%)	0.041
P^c^	< 0.001	0.001	
Glycemic control (HbA1c ≤ 6.5)			
Baseline	19 (26%)	29 (29%)	0.734
Postoperative	57 (79.2%)	84 (84%)	0.429
P^c^	< 0.001	< 0.001	

RYGB: Roux-Y gastric bypass, SG: sleeve gastrectomy, BMI: Body mass index, ^a^Chi^2^, Mann–Whitney U test, ^b^Wilcoxon signed rank test, ^c^McNemar test, ^#^compared with baseline *1 year after surgery, across all severity categories.

**Table 4:** Weight loss according to surgical technique.

	RYGB	SG	*P*^a^
BMI, kg/m^2^			
Baseline	40.7 (IQR 10.6)	45.2 (IQR 12.7)	0.006
Postoperative	30.0 (IQR 8.7)	34.0 (IQR 11.3)	0.009
Change	10.5 (IQR 5.2)	9.8 (IQR 5.4)	0.94
P^b^	< 0.001	< 0.001	
TWL, %	25.5 (IQR 13.2)	23.7 (IQR 10.1)	0.366
EBMIL, %	69.4 (IQR 34.0)	53.2 (IQR 36.0)	0.016

RYGB: Roux-Y gastric bypass, SG: sleeve gastrectomy, BMI: Body mass index, ^a^Mann-Whitney U test, ^b^Wilcoxon signed rank test *1 year after surgery, across all severity categories.

When diabetic remission rates were observed according to disease severity as stratified by the IMS score comparing LRYGB and LSG, they were at 85% and 100% respectively for the mild T2DM category, 60% and 55% for the moderate T2DM category, 18% and 8% for the severe T2DM category (Table 5). Statistically, neither surgical technique displayed any advantage above the other in inducing disease remission within each of the severity categories.

**Table 5:** Diabetic remission rates according to disease severity and surgical technique.

Diabetic severity	RYGB	SG	P*
Mild	17 (85%)	22 (100%)	0.099
Moderate	18 (60%)	36 (55%)	0.662
Severe	4 (18%)	1 (8%)	0.635

*Chi^2^.

When the remission rates and diabetic outcome were analysed between diabetic severity groups, it was found that remission rates in mild disease were the best at 92.9%, compared to 56.2% in moderate and 14.7% in severe disease (*p* < 0.001). This shows that mild disease classified according to the IMS score is more likely to result in diabetic remission after surgery. Despite lower remission rates, 58.8% of severe T2DM patients who underwent BMS still experienced disease improvement (Table 6). Nevertheless, the amount of weight loss was not differential from the diabetic remission rates when compared between disease severity categories within their respective surgical techniques (Table 7).

**Table 6:** Diabetic outcomes 1 year after surgery according to disease severity.

IMS score	Mild	Moderate	Severe	P*
Diabetic outcome				
Remission	39 (92.9%)	54 (56.2%)	5 (14.7%)	< 0.001
Improved	2 (4.8%)	31 (32.3%)	20 (58.8%)	
Unchanged	1 (2.4%)	11 (11.5%)	9 (26.5%)	

* Chi^2^.

**Table 7:** Observation of weight loss according to disease severity within individual surgical techniques.

	BMI Change from baseline	%TWL	%EBMIL
RYGB			
Mild	11.7 (IQR 10.0)	27.9 (IQR 16.3)	64.0 (IQR 29.8)
Moderate	9.6 (IQR 6.3)	22.8 (IQR 15.0)	68.3 (IQR 36.2)
Severe	10.1 (IQR 3.7)	27.2 (IQR 12.8)	79.4 (IQR 43.3)
P^a^	0.397	0.629	0.303
SG			
Mild	9.9 (IQR 5.4)	23.8 (IQR 10.6)	56.8 (IQR 37.2)
Moderate	9.7 (IQR 5.4)	23.8 (IQR 9.9)	52.7 (IQR 35.7)
Severe	11.0 (IQR 9.5)	22.7 (IQR 22.7)	53.5 (IQR 37.2)
P^a^	0.975	0.966	0.925

^a^Kruskal–Wallis test.

## Discussion

In our study which consists of the Southeast Asian population, it has been shown that this subset of individuals is at an increased risk of developing cardiovascular disease and Type 2 Diabetes Mellitus (T2DM) at a lower BMI when compared to other ethnic groups. Age and sex-adjusted risk association for T2DM occurs at a BMI of 30 kg/m^2^ in the Caucasian population, versus 23.9 kg/m^2^ in the South Asian and 26.9 kg/m^2^ in the Chinese population. Southeast Asians also experience T2DM at a lower waist circumference than the Caucasian population, which is likely due to the elevated percentage of body fat and visceral adipose tissues^6^.

Interestingly, our LSG patients tended to be of higher BMI than RYGB, despite not achieving statistical significance. This is because, in our local setting, LSG is the preferred metabolic procedure by surgeons hence the number of patients undergoing LSG is much higher than those undergoing RYGB. Patients within the RYGB group had a longer duration of diabetes, which correlated with a higher percentage of them requiring insulin, as well as a higher number of anti-diabetic medications. Despite this, there were no statistical differences in diabetic outcomes between both groups postoperatively.

Both LSG and LRYGB produced similarly favourable diabetic outcomes. This study validates the results in the current literature in Western countries^7^. In this study, LSG even did slightly better than LRYGB in diabetic remission rates and improving glycemic control. LSG is a procedure that has a shorter learning curve with a shorter duration of surgery and less perioperative risk^8,9^. LSG was found to be safer with fewer complications and provided other metabolic benefits that were superior to LRYGB such as a reduction in atherosclerotic risk and improvement in fatty liver disease^10^. A study on short-term complications following LRYGB and LSG showed that LSG had half the risk of leaks and morbidity and a significantly lower risk of death in the first 30 days^11^. LRYGB may also be complicated by marginal ulceration in rates from 1 to 16%^12^.

Nutritional deficiency after metabolic surgery is more commonly seen after RYGB^13^. Anemia, iron deficiency, vitamin B12 deficiency were all found to be at a higher incidence after RYGB during follow up. Only folate and vitamin D deficiency were more frequent after LSG when compared to RYGB. Some authors even advocate vitamin B12 supplementation in patients after RYGB^14,15^.

In our local setting, we face the lack of specialized bariatric centres, with many patients having logistic issues, which in turn causes high attrition rates and difficulty in follow-up. Poor health awareness by individuals also leads to a paucity of follow-up, allowing for complications such as malnutrition and marginal ulcers. All these factors then favour LSG as the more sensible procedure in our population. Nevertheless, some studies still show that LRYGB produces longer-lasting and more pronounced metabolic effects than LSG^16^. In a large cohort study with five-year follow-up, patients who underwent RYGB showed higher diabetes remission rates and better glycemic control, in stark contrast to the results of multiple randomized controlled trials^17^.

Limitation of our study include the design of this study which is a retrospective observational study, with a small number of patients included. Our study is also limited to results for one year, hence the follow-up may still be too short to reveal any long-term difference between the two procedures. Future studies will need to be performed to assess if the outcomes are sustained beyond one year.

There are several theories whereby the act of losing weight would actually result in diabetic remission or improvement^18^. However, while there is a clear difference in remission rates among severity categories, statistical analysis of markers of weight loss between these categories failed to show any clear difference. Several mechanisms which occur after LSG and RYGB are likely accountable for diabetes remission. A crucial component is the modulation of gastrointestinal hormones such as glucagon-like peptide 1, peptide YY, cholecystokinin, ghrelin, glucagon, obestatin and oxyntomodulin. These hormones are implicated in energy and glucose homeostasis and are likely responsible for the early metabolic improvements which occur before weight loss^19^.

The IMS score was not useful for procedural selection in obese Malaysians with T2DM undergoing BMS, based on the T2DM remission rates at 1-year post-surgery. However, the IMS score was found to be useful in predicting T2DM remission. It appears that with milder T2DM, the chance for remission is higher. This is likely due to the patient having more functioning pancreatic beta cells for endogenous insulin secretion. The metabolic benefits of early bariatric surgery could prevent or delay the onset of diabetic complications before they become irreversible^20,21^.

Now that a cohort of T2DM obese patients has been identified in our bariatric centre, further studies on long-term diabetic performance in LRYGB and LSG can be performed. However, the limitations of being a single-centre study are that the results may not apply to other centres in the country. Other issues that should be considered are postoperative morbidity and mortality, quality of life, length of hospital stay and costs involved for each of the procedures.

## Conclusion

For obese and diabetic Malaysian patients, LSG is not inferior to LRYGB in terms of T2DM outcomes at one year. However, LRYGB patients show significantly better weight loss. BMS should be considered as an early treatment option for obese patients with T2DM to prevent metabolic complications. In obese patients with T2DM, LSG should be considered a first-line option because of similar diabetic outcomes, its lower perioperative risks, and it is the simpler procedure, except for those with severe gastroesophageal reflux disease.

## Methodology

This is a retrospective observational study reviewing differences in diabetic outcomes of LRYGB and LSG in obese Malaysians with T2DM at 1 year following surgery. A total of 209 consecutive patients who have undergone bariatric and metabolic surgery at Hospital Canselor Tuanku Muhriz of the National University of Malaysia from 1st January 2012 to 31st July 2019 were identified through electronic medical records.

The study was conducted following the principles of the Declaration of Helsinki, and was approved by our institutional ethics review board, Universiti Kebangsaan Malaysia Research Ethics Committee (reference number: JEP-2019-431). This study was also registered with the Malaysia National Medical Research Register with the number NMRR-19-1295-47841. Written informed consent was taken before study commencement. All data collected were kept confidential, and patients were allowed to refuse participation in the study. The data presented do not identify individuals.

All obese patients above 18 years of age with T2DM who underwent either LRYGB or LSG were included. Patients who had revision surgery, had incomplete data, defaulted follow-up or died within 1 year of index surgery were excluded from our analysis. A total of 37 patients were excluded. 12 patients had defaulted follow-up before 1 year, and 24 patients did not have the required data. 1 patient underwent revision surgery within 1 year of index surgery. There was no mortality among the studied patients within 1 year of surgery**.** We resulted in 172 patients that composed the final study population. The patients were then stratified according to the IMS score for analysis.

We diagnosed T2DM by a fasting blood sugar value of ≥ 7.0 mmol/L or an HbA1c level of ≥ 6.3%. The targets for adequate glycemic control are a fasting blood sugar value is 4.4–7.0 mmol/L or an HbA1c level of ≤ 6.5%^4^. We have applied the definition of diabetic remission by the American Society for Bariatric and Metabolic Surgery as having an HbA1c level ≤ 6.4% without taking any anti-diabetic medications for a year. Patients who do not meet the criteria for remission but have their medications reduced by one type or dosage reduced by half are considered to be having disease improvement, whereas those who did not meet any of the aforementioned criteria are considered unchanged^22,23^.

The Individualized Metabolic Surgery (IMS) score was originally devised by Aminian et al. to aid procedural selection between RYGB and SG in obese T2DM patients. This score is also helpful to our study to stratify the patients into the three diabetic severity categories, and the recommended surgical treatment for each category. For the mild disease, LRYGB was suggested due to its advantages over LSG in long-term T2DM remission. For severe diabetes, LSG was suggested as although both procedures resulted in equally low T2DM remission rates, LSG still had an overall better risk–benefit ratio. However, for the moderate severity group, LRYGB was recommended as it appeared to be significantly more effective than LSG^24^.

LRYGB is performed in our centre by fashioning 100 cm of the bilio-pancreatic limb and 100–200 cm of the alimentary limb. The gastric pouch is approximately 50mls and the gastrojejunostomy is created by a stapler technique with an anastomosis 1.2 cm in diameter. LSG is performed by resecting the stomach fundus and corpus over a size 36Fr bougie and leaving a 4 cm long antrum. The gastric remnant volume is about 120 ml.

### Statistical analysis

Statistical analysis was performed using SPSS version 22.0(SPSS Inc., Chicago, IL.). The normality of the continuous variables was examined using the SPSS software. Only the age was normally distributed and described using the mean with standard deviation. Other continuous parameters were not normally distributed and were expressed using the median with the interquartile range. Categorical variables were presented using the count and percentage.

For the comparison of non-normally distributed continuous variables between RYGB and SG groups, the non-parametric Mann Whitney U test was used. This includes the number of diabetic medications, duration of T2DM, preoperative and postoperative HbA1c levels, BMI, total weight loss and excess BMI loss. The normally distributed age was compared using the independent samples t-test. χ2 tests were used for the categorical data, which included the race distribution, T2DM severity categories distribution, diabetic remission rates, glycemic control rates, insulin use rates, and number not taking diabetic medications.

All continuous preoperative data were compared with the postoperative results using Wilcoxon signed-rank tests, whereas categorical performance measures were compared using McNemar tests. Performance measures of weight loss (change in BMI, TWL and EBMIL) within each surgical technique were analysed between diabetic severity categories using the Kruskal–Wallis test (Supplementary Information). A 2-sided *p* value of 0.05 was considered statistically significant.

### Supplementary Information

Supplementary Information 1.

## Data Availability

The datasets used and analysed during the current study are available from the corresponding author on reasonable request.
